# Economic Burden of Pediatric Asthma: Annual Cost of Disease in Iran

**Published:** 2018-02

**Authors:** Laleh SHARIFI, Raheleh DASHTI, Zahra POURPAK, Mohammad Reza FAZLOLLAHI, Masoud MOVAHEDI, Zahra CHAVOSHZADEH, Habib SOHEILI, Saied BOKAIE, Anoushiravan KAZEMNEJAD, Mostafa MOIN

**Affiliations:** 1. Immunology, Asthma and Allergy Research Institute, Tehran University of Medical Sciences, Tehran, Iran; 2. Uro-Oncology Research Center, Tehran University of Medical Sciences, Tehran, Iran; 3. Dept. of Allergy and Clinical Immunology, Children’s Medical Center, Tehran University of Medical Sciences, Tehran, Iran; 4. Pediatric Infectious Research Center, Mofid Children Hospital, Shahid Beheshti University of Medical Sciences, Tehran, Iran; 5. Dept. of Allergy and Immunology, Amirkabir Hospital, Arak University of Medical Sciences, Arak, Iran; 6. Dept. of Epidemiology, Faculty of Veterinary Medicine, University of Tehran, Tehran, Iran; 7. Dept. of Biostatistics, School of Medical Sciences, Tarbiat Modares University, Tehran, Iran

**Keywords:** Asthma, Economic burden, Direct cost, Indirect cost

## Abstract

**Background:**

Asthma is the first cause of children hospitalization and need for emergency and impose high economic burden on the families and governments. We aimed to investigate the economic burden of pediatric asthma and its contribution to family health budget in Iran.

**Methods:**

Overall, 283 pediatric asthmatic patients, who referred to two tertiary pediatric referral centers in Tehran capital of Iran, included from 2010–2012. Direct and indirect asthma-related costs were recorded during one-year period. Data were statistically analyzed for finding association between the costs and factors that affect this cost (demographic variables, tobacco smoke exposure, control status of asthma and asthma concomitant diseases).

**Results:**

Ninety-two (32.5%) females and 191(67.5%) males with the age range of 1–16 yr old were included. We found the annual total pediatrics asthma related costs were 367.97±23.06 USD. The highest cost belonged to the medications (69%) and the lowest one to the emergency (2%). We noticed a significant increasing in boys’ total costs (*P*=0.011), and 7–11 yr old age group (*P*=0.018). In addition, we found significant association between total asthma costs and asthma control status (*P*=0.011).

**Conclusion:**

The presence of an asthmatic child can consume nearly half of the health budget of a family. Our results emphasis on improving asthma management programs, which leads to successful control status of the disease and reduction in economic burden of pediatric asthma.

## Introduction

Asthma is a chronic obstructive respiratory disease in all age groups and for both sexes. It happens after an allergen stimulation that leads to severe symptomatic attacks of bronchial constriction and recurrent episodes of coughing, chest tightness, wheezing and breathlessness (1).

There is sharp rise in the prevalence of asthma in many parts of the world with adopted western lifestyle (2). Overall, 300 million asthmatic patients are present around the world with different ethnical backgrounds; 100 million new cases of asthma will be added to this population by 2025 (3).

The world health organization in a study on global burden of diseases estimated 13.8 million disability-adjusted life years (DALYs) per year because of asthma that encompasses 1.8% of total burden of all diseases (4). Asthma disability level is resembled diabetes (5) and it can affect the social, physical and emotional aspects of patients’ lives especially in those with poor control of the situation (6).

Investigation of burden of diseases published by WHO revealed a strong effect of economic aspect of asthma on quality of life of patients and their families (7). Several investigations of economic costs of asthma have been carried out in different developed counties (8, 9) and 1%–2% of health budget of western countries are spent for asthma (5). Besides, there is limited data from developing countries about the economic burden of asthma (6).

Asthma is responsible for death of many children in developed countries (10). A sharp increasing in the prevalence, mortality, morbidity, and economic burden has been reported for pediatric asthma (5, 11). Asthma has substantial value in children due to its first rank in leading cause of admission children to bed and emergency. Moreover, it is the first reason for students' absence from school (12).

The global prevalence of asthma is different by reason of the lack of internationally accepted definition for asthma (13). According to the standardized methods, the prevalence of asthma symptom reported from 1% to 16% in different countries (5, 14). The pediatric asthma symptoms in recent decades show a reduction in Western Europe, but it is increasing in populations that the prevalence was low before such as Africa, Eastern Europe and Latin America (5). Data based on ISAAC III show that prevalence of current asthma in Iran is about 5.4% in children aged 13–14 yr old (14). A systematic review of asthma symptoms in Iran showed the prevalence of 13.14% for people younger than 18 yr old (15). Therefore, asthma related costs in both direct and indirect proportions are high in Iranian children and adversely affects the families and government.

Economic aspect of asthma is important for both asthmatic patients and health administrators of countries for precise allocating the health budget. In studies on asthma economic burden, attention should be paid for both direct (i.e. medication, doctor visit, emergency services, hospitalization) and indirect costs (i.e. loss of productivity and premature death) (16, 17).

In our previous study (18) on the economic burden, we reported annual asthma cost almost 590 USD in adult asthmatic patients. However, according to increasing prevalence of pediatric asthma in Iran and subsequently its high medical and non-medical costs, we designed a prospective approach to investigate the costs of asthma in Iranian children. Furthermore, in this study we aimed to search on the effect of asthma determinants like tobacco smoke exposure, asthma concomitant diseases, and control status of asthma as well as demographic variables on the economic burden of pediatric asthma in Iran.

## Methods

Overall, 283 asthmatic children entered this prospective study. The patients referred to Children Medical Center and Mofid Hospital, Tehran, Iran as the most important tertiary referral centers of pediatrics in Iran. The inclusion criteria for patients was the age ≤16 yr, diagnosis of asthma by pediatric allergist and immunologist based on GINA 2009 guideline (1), consistent use of asthma medications and regular visit of patients in the above mentioned medical centers. Patients with other respiratory diseases excluded from the study.

This study was confirmed by Ethical Committee of Asthma and Allergy Research Institute, Tehran University of Medical Sciences and all the patients filled the informed consent before participating in the study.

The patients entered the study during Jun 2010 to Jun 2012, for documentation of their asthma related costs and control situation of asthma. Asthma costs categorized into indirect and direct costs recorded in a questionnaire that filled out by trained physicians.

The first part of questionnaire was consisted of questions about demographic variables (i.e., age, sex, smoking status (passive smoker or not), insurance type), the disease history (i.e., asthma comorbid diseases such as sinusitis, allergic rhinitis, nasal polyp, gastroesophageal reflux disease (GERD)) and asthma control status (i.e., complete controlled, partial controlled and uncontrolled). The costs of asthma recorded regularly during one-year follow-up period in second part of questionnaire. The recorded direct costs consisted of doctor visit, asthma medication, radiology, laboratory, skin prick test, spirometry, emergency, hospital admission. The only indirect assessed cost of asthma was transportation. The entire recorded price in questionnaire was as Rial for convenience. All the expenditure were converted to US Dollar based on the exchange data of the Iran Central Bank (19).

The data of this study were analyzed statistically by SPSS software version 19 (Chicago, IL, USA). We used standard statistical descriptions such as mean, minimum, maximum, and standard error for each cost. Association of asthma economic costs according to age, sex, asthma control status, comorbidities and smoking status were investigated. Independent samples *t*-test and one way ANOVA and post hoc test, Tukey were used to compare means of expenditures. The *P*-values less than 0.05 considered statistically significant.

## Results

Among included asthmatic pediatric patients, 283 patients had successful one-year period follow-up; consisting of 92 (32.5%) females and 191 (67.5%) males, with the age range of 1–16 yr old (mean±SE=6.91±0.18). Only 33(11.7%) patients did not have any health insurance whereas 250 (88.3%) had health insurance. Assessment of parental education level showed that 118 (41.7%) of fathers had a university degree and 124 (43.8%) had high school diploma and others did not complete the high school. Whereas 132 (46.6%) mothers were graduated from university, 109 (38.5) mothers had high school diploma and the rest of mothers were educated less than high school. Patients had 1 to 34 d absent from school due to asthma problems during one year (mean±SE=1.7±0.21). Patients during one-year period had 1–18 times doctor visit (mean±SE= 4.44±0.18), they needed to emergency bed 0–3 times (mean±SE= 0.07±0.02), hospital bed day 0–11 times (mean±SE= 0.17±0.06). Patients performed X-ray radiography, skin prick test, laboratory test and spirometry, 0.31±0.03, 0.25±0.49, 0.32±0.03, 0.41±0.04 times respectively during this period.

**Fig. 1:**
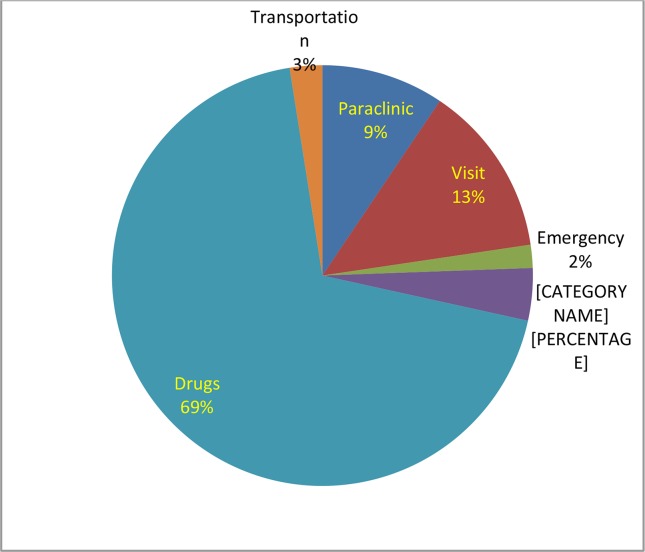
Distribution of pediatric asthma related costs during one-year period in Iran

We estimated total pediatrics asthma related costs for one-year period as 367.97±23.06 USD. The details of expenditures demonstrated in (Fig. 1).

Table 1 and 2 show distributions of total costs in different age and sex groups, ANOVA statistical test showed significant increasing in the total asthma cost of boys comparing girls (*P*=0.011). In addition, we found significant association between total asthma cost in different age groups (*P*=0.008), Tukey HSD revealed that 7–11 yr old asthmatic children have higher asthma related costs than <7 yr old group (*P*=0.018).

**Table 1:** Annual direct and indirect costs of Pediatric asthma in Iran

***Annual costs***		***Minimum***	***Maximum***	***Mean***	***Std. error***
Direct	X-ray	0.00	99.62	3.92	0.69
	Skin Prick Test	0.00	163.38	7.98	1.34
	Laboratory tests	0.00	557.88	16.44	3.24
	Spirometry	0.00	199.24	6.17	1.04
	Visits	5.06	1004.58	48.46	4.99
	Emergency admition	0.00	358.64	6.49	2.40
	Hospitalization	0.00	1235.31	14.74	6.85
	Drugs	1.99	2558.28	252.94	17.30
Indirect	Transportation	0.00	199.24	9.15	1.35

**Table 2:** Annual total asthma costs according to sex, age, and smoking status

***Variable***		***Minimum***	***Maximum***	***Mean***	***Std. error***	***P-value***
Sex	Male	9.71	2575.61	407.44	30.87	0.011
	Female	7.97	1717.67	285.52	28.97	
Age(yr)	<7	7.97 ^a*^	2575.61	305.07	28.45	0.008
	7–11*	22.12 ^bc^	2027.10	396.80	34.62	
	>11*	12.15 ^c^	2575.61	367.97	23.05	
Smoking status	Non-smoker	9.71	2575.61	350.26	25.40	0.14
	Passive smoker	7.97	2027.10	433.70	53.54	

* Common Characters indicate none significant differences with each other.

**Fig. 2:**
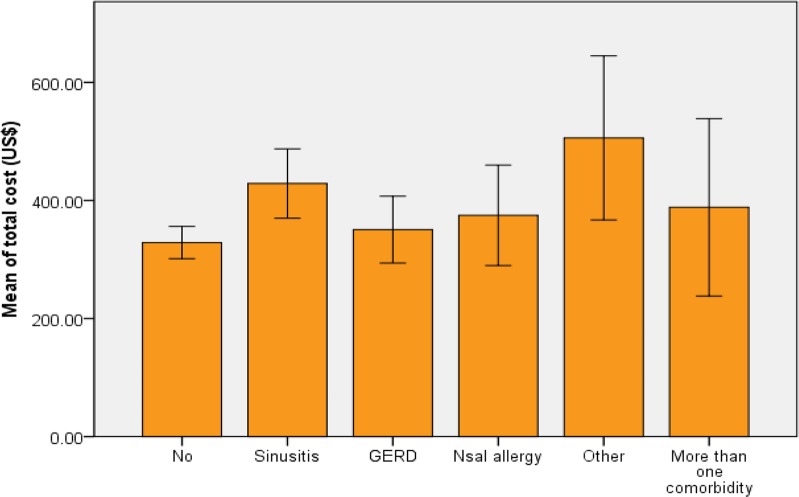
Mean and standard error of total asthma related costs based on asthma co-morbid diseases

**Fig. 3:**
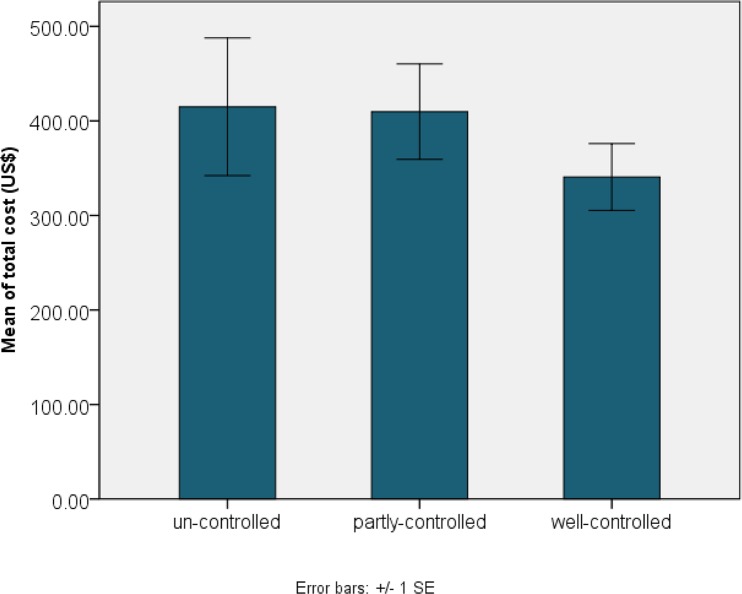
Mean and standard error of total asthma related costs based on control status of asthma

Sixty (20.2%) of asthmatic children had smoker parents and 223(78.8%) had non-smoker parents (*P*=0.14). Almost half of patients, 150(53.0%) had not any comorbid diseases of asthma but 55(19.4%) had sinusitis, 25(8.8%) nasal allergy, 24(8.5%) GERD and the rest of patients had other concomitant disease or combination of comorbidities, statistical analysis did not show any significant difference between total cost of these categories (Fig. 2, *P*=0.16). Asthma control assessment showed that 22(11.0%) of patients had un-controlled asthma, 73(33.3%) had partly controlled and 122(55.7%) had well-controlled asthma. Comparing the means of different control groups showed significant association between control status of asthma and total asthma related costs (Fig. 3, *P*=0.011), and Post Hoc test showed that this association is related to significant differences between partly-controlled and un-controlled groups (*P*=0.047).

## Discussion

We designed a prospective approach to investigate the costs of pediatric asthma in Iran and the factors that affect this cost. Average of annual cost for one pediatric asthma patient is 368 USD, additionally we found a substantial relation between age, sex, and control status of asthma with children asthma costs.

In 2010, Iran Statistics Center published Urban and Rural Household Income and Expenditure indicates that a normal Iranian household expends about 785 USD for health care. This amount is near to 8% of all annual expenditure (20). According to the calculated total cost of asthma (368 USD), the presence of an asthmatic child can consume nearly half of the health budget and imposes high economic burden to family.

The estimated cost of asthma in variety of countries such as the United States (21, 22), Canada (23), the United Kingdom (24), and Italy (25) show a significant quantity moreover results from few economic studies in developing countries show that asthma impose a high economic burden in these countries (6). However, the results from several studies on economic burden of asthma showed ranging from 300 to 1300 USD per patient (26–28).

However, our results show differences with previously similar studies in Iran, for example, a study was investigated retrospectively on 72 asthmatic children under 16 yr old and showed that the mean annual cost per child was approximately 466 USD in Iran (29). It seems the reason of dissimilarity between results is related to different sample size and research methods. In addition to our higher sample size, retrospective investigation of disease costs from patients’ medical records is not reliable enough because all disease related costs do not register completely and correctly in patients’ medical files. We tried to fill this gap by our prospective approach for economic data collection in our continuous contact with patients during one-year period.

In this study, the annual emergency cost for a pediatric asthma patient is 6.49 USD, whereas the emergency costs of asthmatic children were reported 15.23 USD (30). However, this value is not comparable with our findings because they found this cost only for patients with acute asthma attack who need emergency services while we investigated average of emergency cost for all asthmatic children not only for those referred to emergency department.

The annual drug cost in our study was 252.94 USD (69% of total costs), which was the highest asthma related cost. The drug cost was the highest one for adult asthmatic patients (55% of total costs) (18). Moreover, the drug cost was the most expensive part of direct costs (17). In Europe in 2003, anti-asthmatic medication was 4.4 billion USD which was 20.3% of total asthma related costs (31) and pharmacy cost is about 20% of total asthma costs of children in the United States (32).

However, medication is the greatest part of asthma treatment regimens, but our results show that medication costs are a bigger proportion (69%) of asthma total costs, which reveals increasing usage of drugs in the therapeutic regimens in Iran. Increased usage of anti-asthmatic drugs maybe relate to poor control situation of asthma in our patients, therefore we investigate the association between total cost of asthma and control situation. We found a significant relationship between total cost of asthma and control situation of asthma (Fig. 3, *P*=0.011).

Multiple comparisons indicate significant difference between total costs of partly controlled patients (388.69±38.39 USD) and well-controlled patients (329.53±38.79 USD). Our finding is in accordance with other studies that show higher economic burden for patients with severe asthma (6, 33). In addition, poor asthma control linked to the higher economic costs of asthma due to the reducing the quality of life and loss of productivity (34). By improving the asthma management program in the country, the economic burden of the disease will reduce. Fortunately, our knowledge is increasing about the effective methods in controlling of asthma and the results of controlled clinical trials show that a good control of asthma can be feasible in most cases (34). For example experiment of The Finnish Asthma Program during 1994–2004 which focused on early intervention and disease control, successfully resulted in reduction of asthma morbidity and subsequent expenditure (35).

Several concomitant diseases are frequently presented with asthma disease especially in cases with poor responses to treatment or severe asthma (36). Concomitant diseases of asthma can affect the asthma related costs, but we did not find any differences of total economic cost of asthma in different groups of patients with comorbidities such as sinusitis, allergic rhinitis, nasal polyp, and gastroesophageal reflux disease (GERD) (*P*=0.16). According to the relation of the literacy and economic status of families as well as access to medical facilities, we investigated the parents’ education level and economic burden of pediatric asthma but our data did not support any significant relation.

Although prenatally or after birth exposure to cigarette smoke is connected to increased risk of asthma-like symptoms (37), the results of this study did not support the idea that passive tobacco smoking can increase the asthma related costs of children (*P*=0.14).

We show a profound increase in male patients’ costs; asthma related costs of boys was 1.4 times of girls (*P*=0.011). Before age of 14, male sex is a risk factor for asthma (38), smaller size of lung and airways of boys maybe the explanation for this difference (39); however, there is no evidence for the severity of asthma in boys and subsequently their increased asthma costs. Men significantly paid more than women did but no reason for this difference found that maybe it is related to gender discrimination. However, it seems that it is a hypothesis for more sociological researches. In addition, we found significant association between age and total asthma costs (*P*=0.08); Tukey test showed that this difference is related to significant differences of total costs between patients less than 7 yr old and those with 7–11 yr old. Non-specific and variable clinical symptoms of asthma in young children encounter the asthma management with some problems including difficulties in diagnosis and drug efficacy (40) that can result in increasing the asthma related costs for younger children.

The public coverage of health insurance can diminished the intensity of economic burden of asthma for families. Fortunately, in our study, 88.3% of patients had health insurance that shows an improvement in health system of Iran.

The most important limitation in this study was lack of registration of accurate financial data in patients’ health records; however, we tried to fill this gap by continuous contact with patients’ parents for asking about their asthma related expenses. We declare because of parents absentmindedness in some cases, prospective approach is not completely exact method for registration of disease cost.

## Conclusion

Economic burden of pediatric asthma is high in Iran as having an asthmatic child in family can consume half of family health budget. Results are valuable for health administrators as asthma can impose huge economic burden on families and subsequently, its effect on society can be high. In addition, asthma medication accounts for 69% of total costs of asthma. The high proportion most likely are associated with bad control status of patients, therefore, we suggest that upgrading of asthma management programs leads to successful control status of the disease and reduction in economic burden of pediatric asthma. In addition, providing electronic health records for Iranian patients and founding a national center for assessment of medical expenditures in ministry of health will provide easy access to financial information about the diseases and highly recommended.

## Ethical considerations

Ethical issues (Including plagiarism, informed consent, misconduct, data fabrication and/or falsification, double publication and/or submission, redundancy, etc.) have been completely observed by the authors.
